# Supplementary material for: The protein architecture of the endocytic coat analyzed by FRET microscopy
Source: Mol Syst Biol. 2020 May 13;16(5):e9009. doi: 10.15252/msb.20199009 (PMC7218409; doi:10.15252/msb.20199009)
Supplement: Supplementary file 1 — Appendix [file MSB-16-e9009-s001.pdf]

Appendix

The protein architecture of the endocytic coat analyzed by FRET microscopy

Michal Skruzny[1,2,*], Emma Pohl[1,2], Sandina Gnoth[1,2], Gabriele Malengo[1,2], Victor Sourjik[1,2]

Appendix Figure S1 - Partial loss of FRET between Ent1-mTurquoise2 and Sla1-mNeonGreen during endocytic membrane invagination. The second dataset.

FRET values (in %) of 71 or 72 endocytic patches containing or absent of Abp1, respectively, are shown as box plots. Center, top and bottom lines of box plots show the medians, the 25th and 75th percentiles of individual datasets, respectively. Whiskers extend 1.5 times the interquartile range from the 25th and 75th percentiles. Notches indicate 95% confidence intervals. Statistical difference was analyzed by Welch's t-test. See also Figure 4B.

Appendix Figure S2 – Two-hybrid interaction between the N-terminal domain of Chc1 and Ent2-EGFP (top) and Ent1-EGFP (bottom) fusions.

Ent2-/Ent1-GFP fusions isolated from genomes of screened strains were cloned into two-hybrid vectors pGAD-C1 and pTMN38 (pGBD-ENT1), respectively, and cotransformed with indicated plasmids (Collette et al, 2009) into yeast two-hybrid strain pJ964a. The two-hybrid interaction between individual proteins was assessed by the growth on SD -Ura, -Leu, -Ade plates.

Appendix Table S1 - Yeast strains used in this study

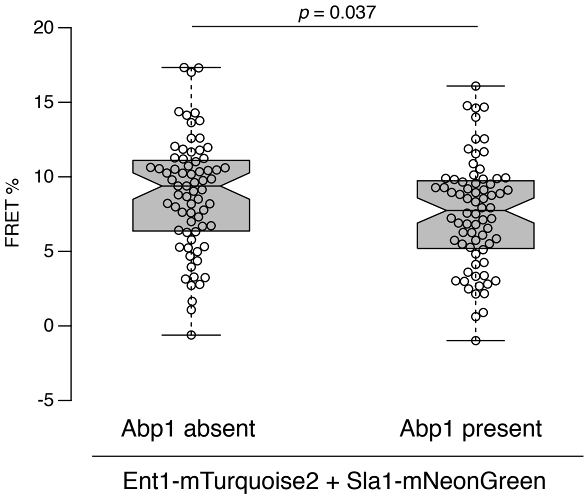

Appendix Figure S1

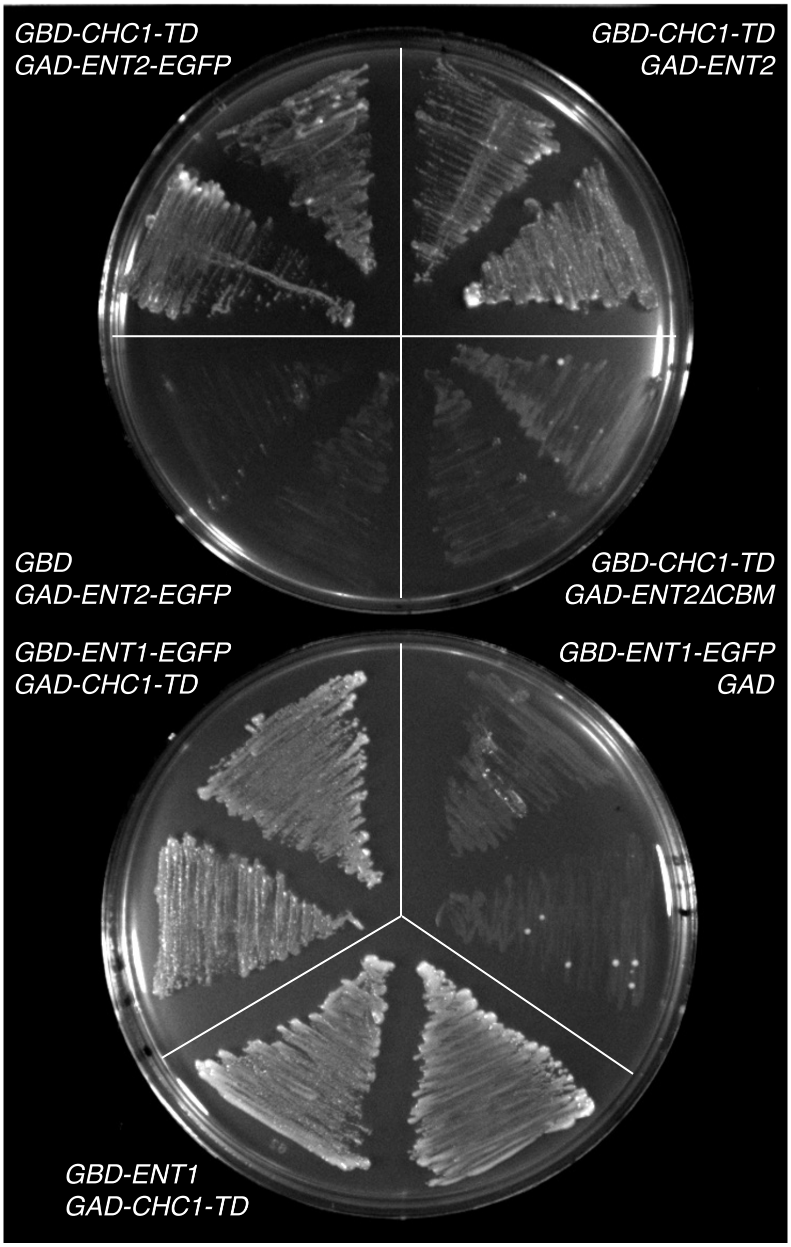

Appendix Figure S2

Appendix Table S1. Yeast Strains used in this study

Strain No.	Short Name	Genotype	Note/Reference
MSK001	wild-type, MATa	*MATa, his3Δ200, leu2-3,112, ura3-52, lys2-801*	S228c background
MSK002	wild-type, MATα	*MATα, his3Δ200, leu2-3,112, ura3-52, lys2-801*	S228c background
MSK004	Sla1-GFP-mCherry	*MATa, ura3-52, leu2Δ1, his3Δ200, trp1Δ63, SLA1-mCherry-sfGFP::kanMX*	Khmelinskii et al., 2012
MSK005	Sla2-GFP-mCherry	*MATa, ura3-52, leu2Δ1, his3Δ200, trp1Δ63, SLA2-mCherry-sfGFP::kanMX*	Khmelinskii et al., 2012
MSK008	GFP-Sla1	*MATα, his3Δ200, GalL-ISceI-nat::leu2-3,112, ura3-52, lys2-801, sfGFP-SLA1*	This Study
MSK009	GFP-Pan1	*MATα, his3Δ200, GalL-ISceI-nat::leu2-3,112, ura3-52, lys2-801, sfGFP-PAN1*	This Study
MSK010	GFP-Sla2	*MATα, his3Δ200, GalL-ISceI-nat::leu2-3,112, ura3-52, lys2-801, sfGFP-SLA2*	Kaksonen Lab
MSK011	Ent1-GFP, Sla1-mCherry	*MATa, his3Δ200, leu2-3,112, ura3-52, lys2-801, ENT1-EGFP::HIS3MX6, SLA1-mCherry::kanMX4*	This Study
MSK012	Ent2-GFP, Sla1-mCherry	*MATα, his3Δ200, leu2-3,112, ura3-52, lys2-801, ENT2-EGFP::HIS3MX6, SLA1-mCherry::kanMX4*	This Study
MSK013	Ent1-GFP, Ede1-mCherry	*MATa, his3Δ200, leu2-3,112, ura3-52, lys2-801, ENT1-EGFP::HIS3MX6, EDE1-mCherry::kanMX4*	This Study
MSK014	Ent2-GFP, Ede1-mCherry	*MATa, his3Δ200, leu2-3,112, ura3-52, lys2-801, ENT2-EGFP::HIS3MX6, EDE1-mCherry::kanMX4*	This Study
MSK015	Pan1-GFP, Ede1-mCherry	*MATa, his3Δ200, leu2-3,112, ura3-52, lys2-801, PAN1-EGFP::HIS3MX6, EDE1-mCherry::kanMX4*	This Study
MSK016	Pan1-GFP, Sla1-mCherry	*MATa, his3Δ200, leu2-3,112, ura3-52, lys2-801, PAN1-EGFP::HIS3MX6, SLA1-mCherry::kanMX4*	This Study
MSK017	Syp1-GFP, Ede1-mCherry	*MATa, his3Δ200, leu2-3,112, ura3-52, lys2-801, SYP1-EGFP::HIS3MX6, EDE1-mCherry::kanMX4*	This Study
MSK018	Ede1-GFP, Sla1-mCherry	*MATa, his3Δ200, leu2-3,112, ura3-52, lys2-801, EDE1-EGFP::HIS3MX6, SLA1-mCherry::kanMX4*	This Study
MSK019	Sla2-GFP, Sla1-mCherry	*MATα, his3Δ200, leu2-3,112, ura3-52, lys2-801, SLA2-EGFP::natNT2, SLA1-mCherry::kanMX4*	This Study
MSK020	Sla2-GFP, Ent1-mCherry	*MATα, his3Δ200, leu2-3,112, ura3-52, lys2-801, SLA2-EGFP::natNT2, ENT1-mCherry::kanMX4*	This Study
MSK021	Syp1-GFP, Sla1-mCherry	*MATa, his3Δ200, leu2-3,112, ura3-52, lys2-801, SYP1-EGFP::HIS3MX6, SLA1-mCherry::kanMX4*	This Study
MSK022	Sla2-GFP, Ede1-mCherry	*MATa, his3Δ200, leu2-3,112, ura3-52, lys2-801, SLA2-EGFP::natNT2, EDE1-mCherry::kanMX4*	This Study
MSK023	Yap1801-GFP, Ede1-mCherry	*MATa, his3Δ200, leu2-3,112, ura3-52, lys2-801, YAP1801-EGFP::HIS3MX6, EDE1-mCherry::kanMX4*	This Study
MSK024	Yap1802-GFP, Ede1-mCherry	*MATa, his3Δ200, leu2-3,112, ura3-52, lys2-801, YAP1802-EGFP::HIS3MX6, EDE1-mCherry::kanMX4*	This Study
MSK025	Apl1-GFP, Ede1-mCherry	*MATa, his3Δ200, leu2-3,112, ura3-52, lys2-801, APL1-EGFP::HIS3MX6, EDE1-mCherry::kanMX4*	This Study
MSK045	Sla1-GFP	*MATa, his3Δ200, leu2-3,112, ura3-52, lys2-801, SLA1-EGFP::HIS3MX6*	This Study
MSK047	Ede1-GFP	*MATa, his3Δ200, leu2-3,112, ura3-52, lys2-801, EDE1-EGFP::HIS3MX6*	This Study
MSK050	Syp1-GFP	*MATa, his3Δ200, leu2-3,112, ura3-52, lys2-801, SYP1-EGFP::HIS3MX6*	This Study
MSK052	Yap1802-GFP	*MATa, his3Δ200, leu2-3,112, ura3-52, lys2-801, YAP1802-EGFP::HIS3MX6*	This Study
MSK053	Yap1801-GFP	*MATa, his3Δ200, leu2-3,112, ura3-52, lys2-801, YAP1801-EGFP::HIS3MX6*	This Study
MSK054	Sla2-GFP	*MATα, his3Δ200, leu2-3,112, ura3-52, lys2-801, SLA2-EGFP::HIS3MX6,*	This Study
MSK055	Pan1-GFP	*MATa, his3Δ200, leu2-3,112, ura3-52, lys2-801, PAN1-EGFP::HIS3MX6*	This Study
MSK057	GFP-End3	*MATα, his3Δ200, GalL-ISceI-nat::leu2-3,112, ura3-52, lys2-801, sfGFP-END3*	This Study
MSK058	Las17-GFP	*MATa, his3Δ200, leu2-3,112, ura3-52, lys2-801, LAS17-EGFP::HIS3MX6*	This Study
MSK060	GFP-Las17	*MATα, his3Δ200, GalL-ISceI-nat::leu2-3,112, ura3-52, lys2-801, sfGFP-LAS17*	This Study
MSK063	End3-GFP	*MATα, his3Δ200, leu2-3,112, ura3-52, lys2-801, END3-EGFP::HIS3MX6*	This Study
MSK070	End3-GFP, Sla1-mCherry	*MATα, his3Δ200, leu2-3,112, ura3-52, lys2-801, END3-EGFP::; SLA1-mCherry::*	This Study
MSK071	Syp1-GFP, Sla2-mCherry	*MATa, his3Δ200, leu2-3,112, ura3-52, lys2-801, SYP1-EGFP::HIS3MX6, SLA1-mCherry::kanMX4*	This Study
MSK073	Syp1-GFP, Pan1-mCherry	*MATa, his3Δ200, leu2-3,112, ura3-52, lys2-801, SYP1-EGFP::natNT2, PAN1-mCherry::kanMX4*	This Study
MSM074	Syp1-GFP, End3-mCherry	*MATa, his3Δ200, leu2-3,112,ura3-52, lys2-801, SYP1-EGFP::HIS3MX6, END3-mCherry::hphNT1*	This Study
MSK075	Syp1-GFP, Las17-mCherry	*MATa, his3Δ200, leu2-3,112,ura3-52, lys2-801, SYP1-EGFP::HIS3MX6, LAS17-mCherry::kanMX*	This Study
MSK076	Yap1802-GFP, Ent1-mCherry	*MATa, his3Δ200, leu2-3,112, ura3-52, lys2-801, YAP1802-EGFP::HIS3MX6, ENT1-mCherry::hphNT1*	This Study
MSK077	Yap1802-GFP, Ent2-mCherry	*MATa, his3Δ200, leu2-3,112, ura3-52, lys2-801, YAP1802-EGFP::HIS3MX6, ENT2-mCherry:::hphNT1*	This Study
MSK078	Yap1802-GFP, Sla2-mCherry	*MATa, his3Δ200, leu2-3,112, ura3-52, lys2-801, YAP1802-EGFP::HIS3MX6, SLA2-mCherry::kanMX/:hphNT1*	This Study
MSK079	Yap1802-GFP, End3-mCherry	*MATa, his3Δ200, leu2-3,112, ura3-52, lys2-801, YAP1802-EGFP::HIS3MX6, END3-mCherry:::hphNT1*	This Study
MSK080	Yap1801-GFP, Sla1-mCherry	*MATa, his3Δ200, leu2-3,112, ura3-52, lys2-801, YAP1801-EGFP::natNT2, SLA1-mCherry:::hphNT1*	This Study
MSK081	Yap1801-GFP, Ent1-mCherry	*MATa, his3Δ200, leu2-3,112, ura3-52, lys2-801, YAP1801-EGFP::HIS3MX6, ENT1-mCherry:::hphNT1*	This Study
MSK082	Yap1801-GFP, Ent2-mCherry	*MATa, his3Δ200, leu2-3,112, ura3-52, lys2-801, YAP1801-EGFP::HIS3MX6, ENT2-mCherry:::hphNT1*	This Study
MSK083	Apl1-GFP	*MATα, his3Δ200, leu2-3,112, ura3-52, lys2-801, APL1-EGFP::natNT2*	This Study
MSK084	Yap1801-GFP, End3-mCherry	*MATa, his3Δ200, leu2-3,112, ura3-52, lys2-801, YAP1801-EGFP::HIS3MX6, END3-mCherry:::hphNT1*	This Study
MSK085	Yap1802-GFP, Sla1-mCherry	*MATa, his3Δ200, leu2-3,112, ura3-52, lys2-801, YAP1802-EGFP::natNT2, SLA1-mCherry::kanMX4*	This Study

MSK086	Yap1801-GFP, Pan1-mCherry	*MATa, his3Δ200, leu2-3,112, ura3-52, lys2-801, YAP1801-EGFP::natNT2, PAN1-mCherry::kanMX4*	This Study
MSK087	Yap1802-GFP, Pan1-mCherry	*MATa, his3Δ200, leu2-3,112, ura3-52, lys2-801, YAP1802-EGFP::natNT2, PAN1-mCherry::kanMX4*	This Study
MSK088	Ent1-GFP, Pan1-mCherry	*MATa, his3Δ200, leu2-3,112, ura3-52, lys2-801, ENT1-EGFP::natNT2, PAN1-mCherry::kanMX4*	This Study
MSK089	Ent2-GFP, Pan1-mCherry	*MATa, his3Δ200, leu2-3,112, ura3-52, lys2-801, ENT2-EGFP::natNT2, PAN1-mCherry::kanMX4*	This Study
MSK090	Sla2-GFP, Pan1-mCherry	*MATa, his3Δ200, leu2-3,112, ura3-52, lys2-801, SLA2-EGFP::natNT2, PAN1-mCherry::kanMX4*	This Study
MSK091	End3-GFP, Pan1-mCherry	*MATa, his3Δ200, leu2-3,112, ura3-52, lys2-801, END3-EGFP::natNT2, PAN1-mCherry::kanMX4*	This Study
MSK092	Las17-GFP, Pan1-mCherry	*MATa, his3Δ200, leu2-3,112, ura3-52, lys2-801, LAS17-EGFP::natNT2, PAN1-mCherry::kanMX4*	This Study
MSK093	Lsb3-GFP, Pan1-mCherry	*MATa, his3Δ200, leu2-3,112, ura3-52, lys2-801, LSB3-EGFP::natNT2, PAN1-mCherry::kanMX4*	This Study
MSK094	GFP-Sla1, Ede1-mCherry	*MATα, his3Δ200, GalL-ISceI-nat::leu2-3,112, ura3-52, lys2-801, sfGFP-SLA1, EDE1-mCherry::kanMX*	This Study
MSK095	GFP-Sla1, Ent1-mCherry	*MATα, his3Δ200, GalL-ISceI-nat::leu2-3,112, ura3-52, lys2-801, sfGFP-SLA1, ENT1-mCherry::kanMX*	This Study
MSK097	GFP-Sla1, Sla2-mCherry	*MATα, his3Δ200, GalL-ISceI-nat::leu2-3,112, ura3-52, lys2-801, sfGFP-SLA1, SLA2-mCherry::kanMX*	This Study
MSK098	GFP-Sla1, End3-mCherry	*MATα, his3Δ200, GalL-ISceI-nat::leu2-3,112, ura3-52, lys2-801, sfGFP-SLA1, END3-mCherry::kanMX*	This Study
MSK099	GFP-Sla1, Las17-mCherry	*MATα, his3Δ200, GalL-ISceI-nat::leu2-3,112, ura3-52, lys2-801, sfGFP-SLA1, LAS17-mCherry::kanMX*	This Study
MSK100	Las17-GFP, Ede1-mCherry	*MATa, his3Δ200, leu2-3,112, ura3-52, lys2-801, LAS17-EGFP::HIS3MX6, EDE1-mCherry::kanMX*	This Study
MSK101	Las17-GFP, Ent1-mCherry	*MATa, his3Δ200, leu2-3,112, ura3-52, lys2-801, LAS17-EGFP::HIS3MX6, ENT1-mCherry::kanMX*	This Study
MSK102	Las17-GFP, Ent2-mCherry	*MATa, his3Δ200, leu2-3,112, ura3-52, lys2-801, LAS17-EGFP::HIS3MX6, ENT2-mCherry::kanMX*	This Study
MSK103	Las17-GFP, Sla2-mCherry	*MATa, his3Δ200, leu2-3,112, ura3-52, lys2-801, LAS17-EGFP::HIS3MX6, SLA2-mCherry::kanMX*	This Study
MSK104	GFP-End3, Ede1-mCherry	*MATa, his3Δ200, GalL-ISceI-nat::leu2-3,112, ura3-52, lys2-801, TEF-URA::sfGFP-END3, EDE1-mCherry::kanMX4*	This Study
MSK109	Las17-GFP, End3-mCherry	*MATa, his3Δ200, leu2-3,112, ura3-52, lys2-801, LAS17-EGFP::HIS3MX6, END3-mCherry::kanMX4*	This Study
MSK110	End3-GFP, Ent1-mCherry	*MATα, his3Δ200, leu2-3,112, ura3-52, lys2-801, END3-EGFP::HIS3MX6, ENT1-mCherry::kanMX4*	This Study
MSK112	End3-GFP, Sla2-mCherry	*MATα, his3Δ200, leu2-3,112, ura3-52, lys2-801, END3-EGFP::HIS3MX6, SLA2-mCherry::kanMX4*	This Study
MSK113	End3-GFP, Las17-mCherry	*MATα, his3Δ200, leu2-3,112, ura3-52, lys2-801, END3-EGFP::HIS3MX6, LAS17-mCherry::kanMX4*	This Study
MSK114	End3-GFP, Ede1-mCherry	*MATa, his3Δ200, leu2-3,112, ura3-52, lys2-801, END3-EGFP::natNT2, EDE1-mCherry::kanMX4*	This Study
MSK115	Lsb3-GFP, Ede1-mCherry	*MATa, his3Δ200, leu2-3,112, ura3-52, lys2-801, LSB3-EGFP::natNT2, EDE1-mCherry::kanMX4*	This Study
MSK116	Pan1-GFP, End3-mCherry	*MATa, his3Δ200, leu2-3,112, ura3-52, lys2-801, PAN1-EGFP::HIS3MX6, END3-mCherry::kanMX4*	This Study
MSK117	Pan1-GFP, Sla2-mCherry	*MATa, his3Δ200, leu2-3,112, ura3-52, lys2-801, PAN1-EGFP::HIS3MX6, SLA2-mCherry::kanMX4*	This Study
MSK119	GFP-Sla2, End3-mCherry	*MATα, his3Δ200, GalL-ISceI-nat::leu2-3,112, ura3-52, lys2-801, sfGFP-SLA2, END3-mCherry::hphNT1*	This Study
MSK120	GFP-Sla2, Las17-mCherry	*MATα, his3Δ200, GalL-ISceI-nat::leu2-3,112, ura3-52, lys2-801, sfGFP-SLA2, LAS17-mCherry::hphNT1*	This Study
MSK121	GFP-Sla2, Lsb3-mCherry	*MATα, his3Δ200, GalL-ISceI-nat::leu2-3,112, ura3-52, lys2-801, sfGFP-SLA2, LSB3-mCherry::hphNT1*	This Study
MSK124	GFP-Ede1	*MATα, his3Δ200, GalL-ISceI-nat::leu2-3,112, ura3-52, lys2-801, sfGFP-EDE1*	This Study
MSK126	Yap1801-GFP, Las17-mCherry	*MATa, his3Δ200, leu2-3,112, ura3-52, lys2-801, YAP1801-EGFP::HIS3MX6, LAS17-mCherry:::hphNT1*	This Study
MSK127	Yap1801-GFP, Lsb3-mCherry	*MATa, his3Δ200, leu2-3,112, ura3-52, lys2-801, YAP1801-EGFP::HIS3MX6, LSB3-mCherry::hphNT1*	This Study
MSK128	Yap1802-GFP, Las17-mCherry	*MATa, his3Δ200, leu2-3,112, ura3-52, lys2-801, YAP1802-EGFP::HIS3MX6, LAS17-mCherry:::hphNT1*	This Study
MSK129	Yap1802-GFP, Lsb3-mCherry	*MATa, his3Δ200, leu2-3,112, ura3-52, lys2-801, YAP1802-EGFP::HIS3MX6, LSB3-mCherry::hphNT1*	This Study
MSK130	Ent2-GFP, Sla2-mCherry	*MATa, his3Δ200, leu2-3,112, ura3-52, lys2-801, ENT2-EGFP::HIS3MX6, SLA2-mCherry::kanMX4*	This Study
MSK131	GFP-Ede1, Chc1-mCherry	*MATα, his3Δ200, GalL-ISceI-nat::leu2-3,112, ura3-52, lys2-801, sfGFP-EDE1, CHC1-mCherry::hphNT1*	This Study
MSK132	Ede1-GFP, Chc1-mCherry	*MATa, his3Δ200, leu2-3,112, ura3-52, lys2-801, EDE1-EGFP::HIS3MX6, CHC1-mCherry::hphNT1*	This Study
MSK133	GFP-Sla2, Chc1-mCherry	*MATα, his3Δ200, GalL-ISceI-nat::leu2-3,112, ura3-52, lys2-801, sfGFP-SLA2, CHC1-mCherry::hphNT1*	This Study
MSK134	Sla2-GFP, Chc1-mCherry	*MATa, his3Δ200, leu2-3,112, ura3-52, lys2-801, SLA2-EGFP::HIS3MX6, CHC1-mCherry::hphNT1*	This Study
MSK136	Syp1-GFP, Chc1-mCherry	*MATa, his3Δ200, leu2-3,112, ura3-52, lys2-801, SYP1-EGFP::HIS3MX6, CHC1-mCherry::hphNT1*	This Study
MSK137	GFP-Pan1, Chc1-mCherry	*MATα, his3Δ200, GalL-ISceI-nat::leu2-3,112, ura3-52, lys2-801, sfGFP-PAN1, CHC1-mCherry::hphNT1*	This Study
MSK138	Pan1-GFP, Chc1-mCherry	*MATa, his3Δ200, leu2-3,112, ura3-52, lys2-801, PAN1-EGFP::HIS3MX6, CHC1-mCherry::hphNT1*	This Study
MSK139	Yap1801-GFP, Chc1-mCherry	*MATa, his3Δ200, leu2-3,112, ura3-52, lys2-801, YAP1801-EGFP::HIS3MX6, CHC1-mCherry::hphNT1*	This Study
MSK140	Yap1802-GFP, Chc1-mCherry	*MATa, his3Δ200, leu2-3,112, ura3-52, lys2-801, YAP1802-EGFP::HIS3MX6, CHC1-mCherry::hphNT1*	This Study
MSK141	Ent1-GFP, Chc1-mCherry	*MATα, his3Δ200, leu2-3,112, ura3-52, lys2-801, ENT1-EGFP::HIS3MX6, CHC1-mCherry::hphNT1*	This Study
MSK142	Ent2-GFP, Chc1-mCherry	*MATα, his3Δ200, leu2-3,112, ura3-52, lys2-801, ENT2-EGFP::HIS3MX6, CHC1-mCherry::hphNT1*	This Study
MSK143	GFP-Sla1, Chc1-mCherry	*MATα, his3Δ200, GalL-ISceI-nat::leu2-3,112, ura3-52, lys2-801, sfGFP-SLA1, CHC1-mCherry::hphNT1*	This Study
MSK144	Sla1-GFP, Chc1-mCherry	*MATa, his3Δ200, leu2-3,112, ura3-52, lys2-801, SLA1-EGFP::HIS3MX6, CHC1-mCherry::hphNT1*	This Study
MSK145	GFP-End3, Chc1-mCherry	*MATα, his3Δ200, GalL-ISceI-nat::leu2-3,112, ura3-52, lys2-801, sfGFP-END3, CHC1-mCherry::hphNT1*	This Study
MSK146	End3-GFP, Chc1-mCherry	*MATa, his3Δ200, leu2-3,112, ura3-52, lys2-801, END3-EGFP::HIS3MX6, CHC1-mCherry::hphNT1*	This Study

MSK147	GFP-Las17, Chc1-mCherry	*MATα, his3Δ200, GalL-ISceI-nat::leu2-3,112, ura3-52, lys2-801, sfGFP-LAS17, CHC1-mCherry::hphNT1*	This Study
MSK148	Las17-GFP, Chc1-mCherry	*MATa, his3Δ200, leu2-3,112, ura3-52, lys2-801, LAS17-EGFP::HIS3MX6, CHC1-mCherry::hphNT1*	This Study
MSK149	Lsb3-GFP, Chc1-mCherry	*MATα, his3Δ200, leu2-3,112, ura3-52, lys2-801, LSB3-EGFP::HIS3MX6, CHC1-mCherry::hphNT1*	This Study
MSK150	Syp1-GFP, Clc1-mCherry	*MATa, his3Δ200, leu2-3,112, ura3-52, lys2-801, SYP1-EGFP::HIS3MX6, CLC1-mCherry::hphNT1*	This Study
MSK151	Ede1-GFP, Clc1-mCherry	*MATa, his3Δ200, leu2-3,112, ura3-52, lys2-801, EDE1-EGFP::HIS3MX6, CLC1-mCherry::hphNT1*	This Study
MSK152	GFP-Ede1, Clc1-mCherry	*MATα, his3Δ200, GalL-ISceI-nat::leu2-3,112, ura3-52, lys2-801, sfGFP-EDE1, CLC1-mCherry::hphNT1*	This Study
MSK153	Ent1-GFP, Clc1-mCherry	*MATα his3Δ200, leu2-3,112, ura3-52, lys2-801, ENT1-EGFP::HIS3MX6, CLC1-mCherry::hphNT1*	This Study
MSK154	Ent2-GFP, Clc1-mCherry	*MATα, his3Δ200, leu2-3,112, ura3-52, lys2-801, ENT2-EGFP::HIS3MX6, CLC1-mCherry::hphNT1*	This Study
MSK155	Yap1801-GFP, Clc1-mCherry	*MATa, his3Δ200, leu2-3,112, ura3-52, lys2-801, YAP1801-EGFP::HIS3MX6, CLC1-mCherry::hphNT1*	This Study
MSK156	Yap1802-GFP, Clc1-mCherry	*MATa, his3Δ200, leu2-3,112, ura3-52, lys2-801, YAP1802-EGFP::HIS3MX6, CLC1-mCherry::hphNT1*	This Study
MSK157	Sla2-GFP, Clc1-mCherry	*MATa, his3Δ200, leu2-3,112, ura3-52, lys2-801, SLA2-EGFP::HIS3MX6, CLC1-mCherry::hphNT1*	This Study
MSK158	GFP-Syp1, Clc1-mCherry	*MATα, his3Δ200, GalL-ISceI-nat::leu2-3,112, ura3-52, lys2-801, sfGFP-SYP1, CLC1-mCherry::hphNT1*	This Study
MSK159	Sla1-GFP, Clc1-mCherry	*MATa, his3Δ200, leu2-3,112, ura3-52, lys2-801, SLA1-EGFP::HIS3MX6, CLC1-mCherry::hphNT1*	This Study
MSK160	GFP-Sla1, Clc1-mCherry	*MATα, his3Δ200, GalL-ISceI-nat::leu2-3,112, ura3-52, lys2-801, sfGFP-SLA1, CLC1-mCherry::hphNT1*	This Study
MSK161	End3-GFP, Clc1-mCherry	*MATa, his3Δ200, leu2-3,112, ura3-52, lys2-801, END3-EGFP::HIS3MX6, CLC1-mCherry::hphNT1*	This Study
MSK162	GFP-End3, Clc1-mCherry	*MATα, his3Δ200, GalL-ISceI-nat::leu2-3,112, ura3-52, lys2-801, sfGFP-END3, CLC1-mCherry::hphNT1*	This Study
MSK163	Pan1-GFP, Clc1-mCherry	*MATa, his3Δ200, leu2-3,112, ura3-52, lys2-801, PAN1-EGFP::HIS3MX6, CLC1-mCherry::hphNT1*	This Study
MSK164	GFP-Pan1, Clc1-mCherry	*MATα, his3Δ200, GalL-ISceI-nat::leu2-3,112, ura3-52, lys2-801, sfGFP-PAN1, CLC1-mCherry::hphNT1*	This Study
MSK165	GFP-Las17, Clc1-mCherry	*MATα, his3Δ200, GalL-ISceI-nat::leu2-3,112, ura3-52, lys2-801, sfGFP-LAS17, CLC1-mCherry::hphNT1*	This Study
MSK166	Las17-GFP, Clc1-mCherry	*MATa, his3Δ200, leu2-3,112, ura3-52, lys2-801, LAS17-EGFP::HIS3MX6, CLC1-mCherry::hphNT1*	This Study
MSK167	Lsb3-GFP, Clc1-mCherry	*MATα, his3Δ200, leu2-3,112, ura3-52, lys2-801, LSB3-EGFP::HIS3MX6, CLC1-mCherry::hphNT1*	This Study
MSK168	Ent1-GFP	*MATα, his3Δ200, leu2-3,112, ura3-52, lys2-801, ENT1-EGFP::HIS3MX6*	This Study
MSK169	Ent2-GFP	*MATα, his3Δ200, leu2-3,112, ura3-52, lys2-801, ENT2-EGFP::HIS3MX6*	This Study
MSK170	Lsb3-GFP	*MATα, his3Δ200, leu2-3,112, ura3-52, lys2-801, LSB3-EGFP::HIS3MX6*	This Study
MSK171	Las17-GFP, Lsb3-mCherry	*MATa, his3Δ200, leu2-3,112, ura3-52, lys2-801, LAS17-EGFP::HIS3MX6, LSB3-mCherry::hphNT1*	This Study
MSK173	Lsb3-GFP, Sla1-mCherry	*MATα, his3Δ200, leu2-3,112, ura3-52, lys2-801,LSB3-EGFP::HIS3MX6, SLA1-mCherry::hphNT1*	This Study
MSK174	Sla1-GFP, Lsb3-mCherry	*MATa, his3Δ200, leu2-3,112, ura3-52, lys2-801, SLA1-EGFP::HIS3MX6, LSB3-mCherry::hphNT1*	This Study
MSK175	Sla2-GFP, Lsb3-mCherry	*MATa, his3Δ200, leu2-3,112, ura3-52, lys2-801, SLA2-EGFP::HIS3MX6, LSB3-mCherry::hphNT1*	This Study
MSK176	End3-GFP, Lsb3-mCherry	*MATa, his3Δ200, leu2-3,112, ura3-52, lys2-801, END3-EGFP::HIS3MX6, LSB3-mCherry::hphNT1*	This Study
MSK177	Syp1-GFP, Ent1-mCherry	*MATa, his3Δ200, leu2-3,112, ura3-52, lys2-801, SYP1-EGFP::HIS3MX6, ENT1-mCherry::hphNT1*	This Study
MSK178	Syp1-GFP, Ent2-mCherry	*MATa, his3Δ200, leu2-3,112, ura3-52, lys2-801, SYP1-EGFP::HIS3MX6, ENT2-mCherry::hphNT1*	This Study
MSK179	Syp1-GFP, Lsb3-mCherry	*MATa, his3Δ200, leu2-3,112, ura3-52, lys2-801, SYP1-EGFP::HIS3MX6, LSB3-mCherry::hphNT1*	This Study
MSK180	Ent2-GFP, Ent1-mCherry	*MATα, his3Δ200, leu2-3,112, ura3-52, lys2-801, ENT2-EGFP::HIS3MX6, ENT1-mCherry::hphNT1*	This Study
MSK181	Ent2-GFP, Lsb3-mCherry	*MATα, his3Δ200, leu2-3,112, ura3-52, lys2-801, ENT2-EGFP::HIS3MX6, LSB3-mCherry::hphNT1*	This Study
MSK192	GFP-Sla2, Ede1-mCherry	*MATα, his3Δ200, GalL-ISceI-nat::leu2-3,112, ura3-52, lys2-801, sfGFP-SLA2, EDE1-mCherry::hphNT1*	This Study
MSK193	GFP-Sla2, Sla1-mCherry	*MATα, his3Δ200, GalL-ISceI-nat::leu2-3,112, ura3-52, lys2-801, sfGFP-SLA2, SLA1-mCherry::hphNT1*	This Study
MSK194	GFP-Sla2, Ent1-mCherry	*MATα, his3Δ200, GalL-ISceI-nat::leu2-3,112, ura3-52, lys2-801, sfGFP-SLA2, ENT1-mCherry::hphNT1*	This Study
MSK195	GFP-Sla2, Ent2-mCherry	*MATα, his3Δ200, GalL-ISceI-nat::leu2-3,112, ura3-52, lys2-801, sfGFP-SLA2, ENT2-mCherry::hphNT1*	This Study
MSK196	GFP-Sla2, Pan1-mCherry	*MATα, his3Δ200, GalL-ISceI-nat::leu2-3,112, ura3-52, lys2-801, sfGFP-SLA2, PAN1-mCherry::hphNT1*	This Study
MSK198	GFP-Sla1, Pan1-mCherry	*MATα, his3Δ200, GalL-ISceI-nat::leu2-3,112, ura3-52, lys2-801, sfGFP-SLA1, PAN1-mCherry::hphNT1*	This Study
MSK199	GFP-Sla1, Lsb3-mCherry	*MATα, his3Δ200, GalL-ISceI-nat::leu2-3,112, ura3-52, lys2-801, sfGFP-SLA1, LSB3-mCherry::hphNT1*	This Study
MSK200	GFP-Ede1, Ent1-mCherry	*MATα, his3Δ200, GalL-ISceI-nat::leu2-3,112, ura3-52, lys2-801, sfGFP-EDE1, ENT1-mCherry::hphNT1*	This Study
MSK201	GFP-Ede1, Ent2-mCherry	*MATα, his3Δ200, GalL-ISceI-nat::leu2-3,112, ura3-52, lys2-801, sfGFP-EDE1, ENT2-mCherry::hphNT1*	This Study
MSK202	GFP-Ede1, Sla2-mCherry	*MATα, his3Δ200, GalL-ISceI-nat::leu2-3,112, ura3-52, lys2-801, sfGFP-EDE1, SLA2-mCherry::hphNT1*	This Study
MSK203	GFP-Ede1, Lsb3-mCherry	*MATα, his3Δ200, GalL-ISceI-nat::leu2-3,112, ura3-52, lys2-801, sfGFP-EDE1, LSB3-mCherry::hphNT1*	This Study
MSK204	GFP-Ede1, Las17-mCherry	*MATα, his3Δ200, GalL-ISceI-nat::leu2-3,112, ura3-52, lys2-801, sfGFP-EDE1, LAS17-mCherry::hphNT1*	This Study
MSK205	GFP-Ede1, Sla1-mCherry	*MATα, his3Δ200, GalL-ISceI-nat::leu2-3,112, ura3-52, lys2-801, sfGFP-EDE1,SLA1-mCherry::hphNT1*	This Study
MSK206	GFP-Ede1, Pan1-mCherry	*MATα, his3Δ200, GalL-ISceI-nat::leu2-3,112, ura3-52, lys2-801, sfGFP-EDE1, PAN1-mCherry::hphNT1*	This Study
MSK207	GFP-Ede1, End3-mCherry	*MATα, his3Δ200, GalL-ISceI-nat::leu2-3,112, ura3-52, lys2-801, sfGFP-EDE1, END3-mCherry::hphNT1*	This Study
MSK208	GFP-Pan1, Ede1-mCherry	*MATα, his3Δ200, GalL-ISceI-nat::leu2-3,112, ura3-52, lys2-801, sfGFP-PAN1, EDE1-mCherry::kanMX4*	This Study

MSK209	GFP-Pan1, Sla2-mCherry	*MATα, his3Δ200, GalL-ISceI-nat::leu2-3,112, ura3-52, lys2-801, sfGFP-PAN1, SLA2-mCherry::kanMX4*	This Study
MSK210	GFP-Pan1, End3-mCherry	*MATα, his3Δ200, GalL-ISceI-nat::leu2-3,112, ura3-52, lys2-801, sfGFP-PAN1, END3-mCherry::kanMX4*	This Study
MSK211	GFP-Pan1, Ent1-mCherry	*MATα, his3Δ200, GalL-ISceI-nat::leu2-3,112, ura3-52, lys2-801, sfGFP-PAN1, ENT1-mCherry::hphNT1*	This Study
MSK212	GFP-Pan1, Ent2-mCherry	*MATα, his3Δ200, GalL-ISceI-nat::leu2-3,112, ura3-52, lys2-801, sfGFP-PAN1, ENT2-mCherry::hphNT1*	This Study
MSK213	GFP-Pan1, Sla1-mCherry	*MATα, his3Δ200, GalL-ISceI-nat::leu2-3,112, ura3-52, lys2-801, sfGFP-PAN1, SLA1-mCherry::hphNT1*	This Study
MSK214	GFP-Pan1, Las17-mCherry	*MATα, his3Δ200, GalL-ISceI-nat::leu2-3,112, ura3-52, lys2-801, sfGFP-PAN1, LAS17-mCherry::hphNT1*	This Study
MSK215	GFP-Pan1, Lsb3-mCherry	*MATα, his3Δ200, GalL-ISceI-nat::leu2-3,112, ura3-52, lys2-801, sfGFP-PAN1, LSB3-mCherry::hphNT1*	This Study
MSK216	GFP-End3, Sla2-mCherry	*MATα, his3Δ200, GalL-ISceI-nat::leu2-3,112, ura3-52, lys2-801, sfGFP-END3, SLA2-mCherry::hphNT1*	This Study
MSK217	GFP-End3, Pan1-mCherry	*MATα, his3Δ200, GalL-ISceI-nat::leu2-3,112, ura3-52, lys2-801, sfGFP-END3, PAN1-mCherry::hphNT1*	This Study
MSK218	GFP-End3, Sla1-mCherry	*MATα, his3Δ200, GalL-ISceI-nat::leu2-3,112, ura3-52, lys2-801, sfGFP-END3, SLA1-mCherry::hphNT1*	This Study
MSK219	GFP-End3, Lsb3-mCherry	*MATα, his3Δ200, GalL-ISceI-nat::leu2-3,112, ura3-52, lys2-801, sfGFP-END3, LSB3-mCherry::hphNT1*	This Study
MSK220	GFP-Las17, Ede1-mCherry	*MATα, his3Δ200, GalL-ISceI-nat::leu2-3,112, ura3-52, lys2-801, sfGFP-LAS17, EDE1-mCherry::kanMX4*	This Study
MSK222	GFP-Las17, End3-mCherry	*MATα his3Δ200, GalL-ISceI-nat::leu2-3,112, ura3-52, lys2-801, sfGFP-LAS17, END3-mCherry::kanMX4*	This Study
MSK223	GFP-Las17, Sla2-mCherry	*MATα, his3Δ200, GalL-ISceI-nat::leu2-3,112, ura3-52, lys2-801, sfGFP-LAS17, SLA2-mCherry::kanMX4*	This Study
MSK224	GFP-Las17, Sla1-mCherry	*MATα, his3Δ200, GalL-ISceI-nat::leu2-3,112, ura3-52, lys2-801, sfGFP-LAS17, SLA1-mCherry::hphNT1*	This Study
MSK225	GFP-Las17, Ent1-mCherry	*MATα, his3Δ200, GalL-ISceI-nat::leu2-3,112, ura3-52, lys2-801, sfGFP-LAS17, ENT1-mCherry::hphNT1*	This Study
MSK226	GFP-Las17, Ent2-mCherry	*MATα, his3Δ200, GalL-ISceI-nat::leu2-3,112, ura3-52, lys2-801, sfGFP-LAS17, ENT2-mCherry::hphNT1*	This Study
MSK227	GFP-Las17, Pan1-mCherry	*MATα, his3Δ200, GalL-ISceI-nat::leu2-3,112, ura3-52, lys2-801, sfGFP-LAS17, PAN1-mCherry::hphNT1*	This Study
MSK228	GFP-Las17, Lsb3-mCherry	*MATα, his3Δ200, GalL-ISceI-nat::leu2-3,112, ura3-52, lys2-801, sfGFP-LAS17, LSB3-mCherry::hphNT1*	This Study
MSK241	Ent1-GFP, End3-mCherry	*MATα, his3Δ200, leu2-3,112, ura3-52, lys2-801, ENT1-EGFP::HIS3MX6, END3-mCherry::hphNT1*	This Study
MSK242	Ent2-GFP, End3-mCherry	*MATα, his3Δ200, leu2-3,112, ura3-52, lys2-801, ENT2-EGFP::HIS3MX6, END3-mCherry::hphNT1*	This Study
MSM243	Sla2-GFP, Pan1(1-1303)-mCherry	*MATα, his3Δ200, leu2-3,112, ura3-52, lys2-801, SLA2-EGFP::HIS3MX6, PAN1(aa1-1303)-mCherry::hphNT1*	This Study
MSK244	Sla2-GFP, Pan1(1-1050)-mCherry	*MATα, his3Δ200, leu2-3,112, ura3-52, lys2-801, SLA2-EGFP::HIS3MX6, PAN1(aa1-1050)-mCherry::hphNT1*	This Study
MSK247	Apl1-GFP, Ent1-mCherry	*MATα, his3Δ200, leu2-3,112, ura3-52, lys2-801, APL1-EGFP::natNT2, ENT1-mCherry::*	This Study
MSK248	Apl1-GFP, Sla2-mCherry	*MATα, his3Δ200, leu2-3,112, ura3-52, lys2-801, APL1-EGFP::natNT2, SLA2-mCherry::*	This Study
MSK249	Apl1-GFP, Pan1-mCherry	*MATα, his3Δ200, leu2-3,112, ura3-52, lys2-801, APL1-EGFP::natNT2, PAN1-mCherry::kanMX4*	This Study
MSK250	Apl1-GFP, Sla1-mCherry	*MATα, his3Δ200, leu2-3,112, ura3-52, lys2-801, APL1-EGFP::natNT2, SLA1-mCherry::kanMX4*	This Study
MSK251	Apl1-GFP, Chc1-mCherry	*MATα, his3Δ200, leu2-3,112, ura3-52, lys2-801, APL1-EGFP::natNT2, CHC1-mCherry::hphNT1*	This Study
MSK252	Apl1-GFP, Clc1-mCherry	*MATα, his3Δ200, leu2-3,112, ura3-52, lys2-801, APL1-EGFP::natNT2, CLC1-mCherry::hphNT1*	This Study
MSK253	Syp1-GFP, Bzz1-mCherry	*MATa, his3Δ200, leu2-3,112, ura3-52, lys2-801, SYP1-EGFP::HIS3MX6, BZZ1-mCherry::hphNT1*	This Study
MSK254	Ede1-GFP, Bzz1-mCherry	*MATa, his3Δ200, leu2-3,112, ura3-52, lys2-801, EDE1-EGFP::HIS3MX6, BZZ1-mCherry::hphNT1*	This Study
MSK255	Pan1-GFP, Bzz1-mCherry	*MATa, his3Δ200, leu2-3,112, ura3-52, lys2-801, PAN1-EGFP::HIS3MX6, BZZ1-mCherry::hphNT1*	This Study
MSK256	Las17-GFP, Bzz1-mCherry	*MATa, his3Δ200, leu2-3,112, ura3-52, lys2-801, LAS17-EGFP::HIS3MX6, BZZ1-mCherry::hphNT1*	This Study
MSK258	GFP-Ede1, Bzz1-mCherry	*MATα, his3Δ200, GalL-ISceI-nat::leu2-3,112, ura3-52, lys2-801, sfGFP-EDE1, BZZ1-mCherry::hphNT1*	This Study
MSK259	GFP-Sla2, Bzz1-mCherry	*MATα, his3Δ200, GalL-ISceI-nat::leu2-3,112, ura3-52, lys2-801, sfGFP-SLA2, BZZ1-mCherry::hphNT1*	This Study
MSK260	GFP-Pan1, Bzz1-mCherry	*MATα, his3Δ200, GalL-ISceI-nat::leu2-3,112, ura3-52, lys2-801, sfGFP-PAN1, BZZ1-mCherry::hphNT1*	This Study
MSK261	GFP-Sla1, Bzz1-mCherry	*MATα, his3Δ200, GalL-ISceI-nat::leu2-3,112, ura3-52, lys2-801, sfGFP-SLA1, BZZ1-mCherry::hphNT1*	This Study
MSK262	GFP-Las17, Bzz1-mCherry	*MATα, his3Δ200, GalL-ISceI-nat::leu2-3,112, ura3-52, lys2-801, sfGFP-LAS17, BZZ1-mCherry::hphNT1*	This Study
MSK263	Sla2-GFP, Bzz1-mCherry	*MATα, his3Δ200, leu2-3,112, ura3-52, lys2-801, SLA2-EGFP::HIS3MX6, BZZ1-mCherry::hphNT1*	This Study
MSK264	Sla2-GFP, Vrp1-mCherry	*MATα, his3Δ200, leu2-3,112, ura3-52, lys2-801, SLA2-EGFP::HIS3MX6, VRP1-mCherry::hphNT1*	This Study
MSK265	GFP-Las17, Vrp1-mCherry	*MATα, his3Δ200, GalL-ISceI-nat::leu2-3,112, ura3-52, lys2-801, sfGFP-LAS17, VRP1-mCherry::hphNT1*	This Study
MSK267	GFP-Sla2, Vrp1-mCherry	*MATα, his3Δ200, GalL-ISceI-nat::leu2-3,112, ura3-52, lys2-801, sfGFP-SLA1, VRP1-mCherry::hphNT1*	This Study
MSK268	GFP-Pan1,Vrp1-mCherry	*MATα, his3Δ200, GalL-ISceI-nat::leu2-3,112, ura3-52, lys2-801, sfGFP-PAN1, VRP1-mCherry::hphNT1*	This Study
MSK269	GFP-Ede1, Vrp1-mCherry	*MATα, his3Δ200, GalL-ISceI-nat::leu2-3,112, ura3-52, lys2-801, sfGFP-EDE1, VRP1-mCherry::hphNT1*	This Study
MSK270	GFP-Sla1, Vrp1-mCherry	*MATα, his3Δ200, GalL-ISceI-nat::leu2-3,112, ura3-52, lys2-801, sfGFP-SLA1, VRP1-mCherry::hphNT1*	This Study
MSK271	Ede1-GFP, Vrp1-mCherry	*MATa, his3Δ200, leu2-3,112, ura3-52, lys2-801, EDE1-EGFP::HIS3MX6, VRP1-mCherry::hphNT1*	This Study
MSK272	Sla1-GFP, Vrp1-mCherry	*MATa, his3Δ200, leu2-3,112, ura3-52, lys2-801, SLA1-EGFP::HIS3MX6, VRP1-mCherry::hphNT1*	This Study
MSK273	Pan1-GFP, Vrp1-mCherry	*MATa, his3Δ200, leu2-3,112, ura3-52, lys2-801, PAN1-EGFP::HIS3MX6, VRP1-mCherry::hphNT1*	This Study
MSK274	Syp1-GFP, Vrp1-mCherry	*MATa, his3Δ200, leu2-3,112, ura3-52, lys2-801, SYP1-EGFP::HIS3MX6, VRP1-mCherry::hphNT1*	This Study
MSK275	Las17-GFP, Vrp1-mCherry	*MATa, his3Δ200, leu2-3,112, ura3-52, lys2-801, LAS17-EGFP::HIS3MX6, VRP1-mCherry::hphNT1*	This Study

MSK292	mTq2-Las17	*MATα, his3Δ200, GalL-ISceI-nat::leu2-3,112, ura3-52, lys2-801, mTurquoise2-LAS17*	This Study
MSK301	mTq2-Las17, mNG-Sla1	*MATα, his3Δ200, GalL-ISceI-nat::leu2-3,112, ura3-52, lys2-801, mTurquoise2-LAS17, mNeonGreen-SLA1*	This Study
MSK306	Pan1(1-1303)-mTq2, Sla2-mNG	*MATα, his3Δ200, leu2-3,112, ura3-52, lys2-801, PAN1(aa 1-1303)-mTurquoise2::URA3, SLA2-mNeonGreen::kanMX4*	This Study
MSK310	Gts1-GFP	*MATα, his3Δ200, leu2-3,112, ura3-52, lys2-801, GTS1-EGFP::HIS3MX6*	This Study
MSK311	Gts1-mNG	*MATα, his3Δ200, leu2-3,112, ura3-52, lys2-801, GTS1-mNeonGreen::kanMX4*	This Study
MSK313	Gts1-GFP, Ent1-mCherry	*MATα, his3Δ200, leu2-3,112, ura3-52, lys2-801, GTS1-EGFP::HIS3MX6, ENT1-mCherry::hphNT1*	This Study
MSK314	Gts1-GFP, Chc1-mCherry	*MATα, his3Δ200, leu2-3,112, ura3-52, lys2-801, GTS1-EGFP::HIS3MX6, CHC1-mCherry::hphNT1*	This Study
MSK315	Gts1-GFP, Clc1-mCherry	*MATα, his3Δ200, leu2-3,112, ura3-52, lys2-801, GTS1-EGFP::HIS3MX6, CLC1-mCherry::hphNT1*	This Study
MSK316	Gts1-GFP, Lsb3-mCherry	*MATα, his3Δ200, leu2-3,112, ura3-52, lys2-801, GTS1-EGFP::HIS3MX6, LSB3-mCherry::hphNT1*	This Study
MSK317	Gts1-GFP, Las17-mCherry	*MATα, his3Δ200, leu2-3,112, ura3-52, lys2-801, GTS1-EGFP::HIS3MX6, LAS17-mCherry::hphNT1*	This Study
MSK318	Gts1-GFP, Ede1-mCherry	*MATα, his3Δ200, leu2-3,112, ura3-52, lys2-801, GTS1-EGFP::HIS3MX6, EDE1-mCherry::hphNT1*	This Study
MSK319	Gts1-GFP, End3-mCherry	*MATα, his3Δ200, leu2-3,112, ura3-52, lys2-801, GTS1-EGFP::HIS3MX6, END3-mCherry::hphNT1*	This Study
MSK320	Gts1-GFP, Bzz1-mCherry	*MATα, his3Δ200, leu2-3,112, ura3-52, lys2-801, GTS1-EGFP::HIS3MX6, BZZ1-mCherry::hphNT1*	This Study
MSK321	Gts1-GFP, Vrp1-mCherry	*MATα, his3Δ200, leu2-3,112, ura3-52, lys2-801, GTS1-EGFP::HIS3MX6, VRP1-mCherry::hphNT1*	This Study
MSK322	Gts1-GFP, Pan1-mCherry	*MATα, his3Δ200, leu2-3,112, ura3-52, lys2-801, GTS1-EGFP::HIS3MX6, PAN1-mCherry::hphNT1*	This Study
MSK323	Gts1-GFP, Sla1-mCherry	*MATα, his3Δ200, leu2-3,112, ura3-52, lys2-801, GTS1-EGFP::HIS3MX6, SLA1-mCherry::hphNT1*	This Study
MSK324	Yap1802-GFP, Gts1-mCherry	*MATα, his3Δ200, leu2-3,112, ura3-52, lys2-801, YAP1802-EGFP::HIS3MX6, GTS1-mCherry::hphNT1*	This Study
MSK325	Apl1-GFP, Gts1-mCherry	*MATα, his3Δ200, leu2-3,112, ura3-52, lys2-801, APL1-EGFP::natNT2, GTS1-mCherry::hphNT1*	This Study
MSK326	End3-GFP, Gts1-mCherry	*MATα, his3Δ200, leu2-3,112, ura3-52, lys2-801, END3-EGFP::HISMX, GTS1-mCherry::hphNT1*	This Study
MSK327	GFP-Ede1, Gts1-mCherry	*MATα, his3Δ200, GalL-ISceI-nat::leu2-3,112, ura3-52, lys2-801, sfGFP-EDE1, GTS1-mCherry::hphNT1*	This Study
MSK328	Ent2-GFP, Gts1-mCherry	*MATα, his3Δ200, leu2-3,112, ura3-52, lys2-801, ENT2-EGFP::HISMX, GTS1-mCherry::hphNT1*	This Study
MSK329	GFP-Pan1, Gts1-mCherry	*MATα, his3Δ200, GalL-ISceI-nat::leu2-3,112, ura3-52, lys2-801, sfGFP-PAN1, GTS1-mCherry::kanMX*	This Study
MSK330	GFP-Sla1, Gts1-mCherry	*MATα, his3Δ200, GalL-ISceI-nat::leu2-3,112, ura3-52, lys2-801, sfGFP-SLA1, GTS1-mCherry::kanMX*	This Study
MSK331	GFP-Las17, Gts1-mCherry	*MATα, his3Δ200, GalL-ISceI-nat::leu2-3,112, ura3-52, lys2-801, sfGFP-LAS17, GTS1-mCherry::kanMX*	This Study
MSK332	GFP-Sla2, Gts1-mCherry	*MATα, his3Δ200, GalL-ISceI-nat::leu2-3,112, ura3-52, lys2-801, sfGFP-SLA2, GTS1-mCherry::kanMX*	This Study
MSK334	GFP-Gts1, Sla2-mCherry	*MATα, his3Δ200, GalL-ISceI-nat::leu2-3,112, ura3-52, lys2-801, sfGFP-GTS1, SLA2-mCherry::hphNT1*	This Study
MSK335	GFP-Gts1, Ent1-mCherry	*MATα, his3Δ200, GalL-ISceI-nat::leu2-3,112, ura3-52, lys2-801, sfGFP-GTS1, ENT1-mCherry::hph1*	This Study
MSK336	GFP-Gts1, Ent2-mCherry	*MATα, his3Δ200, GalL-ISceI-nat::leu2-3,112, ura3-52, lys2-801, sfGFP-GTS1, ENT2-mCherry::hph1*	This Study
MSK337	GFP-Gts1, End3-mCherry	*MATα, his3Δ200, GalL-ISceI-nat::leu2-3,112, ura3-52, lys2-801, sfGFP-GTS1, END3-mCherry::hphNT1*	This Study
MSK338	GFP-Gts1, Sla1-mCherry	*MATα, his3Δ200, GalL-ISceI-nat::leu2-3,112, ura3-52, lys2-801, sfGFP-GTS1, SLA1-mCherry::hphNT1*	This Study
MSK339	GFP-Gts1, Pan1-mCherry	*MATα, his3Δ200, GalL-ISceI-nat::leu2-3,112, ura3-52, lys2-801, sfGFP-GTS1, PAN1-mCherry::hphNT1*	This Study
MSK340	GFP-Gts1, Chc1-mCherry	*MATα, his3Δ200, GalL-ISceI-nat::leu2-3,112, ura3-52, lys2-801, sfGFP-GTS1, CHC1-mCherry::hphNT1*	This Study
MSK341	GFP-Gts1, Clc1-mCherry	*MATα, his3Δ200, GalL-ISceI-nat::leu2-3,112, ura3-52, lys2-801, sfGFP-GTS1, CLC1-mCherry::hphNT1*	This Study
MSK342	GFP-Gts1, Ede1-mCherry	*MATα, his3Δ200, GalL-ISceI-nat::leu2-3,112, ura3-52, lys2-801, sfGFP-GTS1, EDE1-mCherry::hphNT1*	This Study
MSK343	GFP-Gts1, Las17-mCherry	*MATα, his3Δ200, GalL-ISceI-nat::leu2-3,112, ura3-52, lys2-801, sfGFP-GTS1, LAS17-mCherry::hphNT1*	This Study
MSK344	GFP-Gts1, Vrp1-mCherry	*MATα, his3Δ200, GalL-ISceI-nat::leu2-3,112, ura3-52, lys2-801, sfGFP-GTS1, VRP1-mCherry::hphNT1*	This Study
MSK345	GFP-Gts1, Bzz1-mCherry	*MATα, his3Δ200, GalL-ISceI-nat::leu2-3,112, ura3-52, lys2-801, sfGFP-GTS1, BZZ1-mCherry::hphNT1*	This Study
MSK346	GFP-Gts1, Lsb3-mCherry	*MATα, his3Δ200, GalL-ISceI-nat::leu2-3,112, ura3-52, lys2-801, sfGFP-GTS1, LSB3-mCherry::hphNT1*	This Study
MSK348	Yap1801-mNG	*MATα, his3Δ200, leu2-3,112, ura3-52, lys2-801, YAP1801-mNeonGreen::kanMX4*	This Study
MSK349	Bzz1-mNG	*MATα, his3Δ200, leu2-3,112, ura3-52, lys2-801, BZZ1-mNeonGreen::kanMX4*	This Study
MSK350	Apl1-mNG	*MATα, his3Δ200, leu2-3,112, ura3-52, lys2-801, APL1-mNeonGreen::kanMX4*	This Study
MSK352	Ede1-mNG	*MATα, his3Δ200, leu2-3,112, ura3-52, lys2-801, EDE1-mNeonGreen::kanMX4*	This Study
MSK353	Vrp1-mNG	*MATα, his3Δ200, leu2-3,112, ura3-52, lys2-801, VRP1-mNeonGreen::kanMX4*	This Study
MSK359	Las17-mNG	*MATα, his3Δ200, leu2-3,112, ura3-52, lys2-801, LAS17-mNeonGreen::kanMX4*	This Study
MSK360	Lsb3-mNG	*MATα, his3Δ200, leu2-3,112, ura3-52, lys2-801, LSB3-mNeonGreen::kanMX4*	This Study
MSK362	mNG-Sla2, Gts1-mSc	*MATα, his3Δ200, GalL-ISceI-nat::leu2-3,112, ura3-52, lys2-801, mNeonGreen-SLA2, GTS1-mScarlet::hphNT1*	This Study
MSK364	mNG-Pan1, Gts1-mSc	*MATα, his3Δ200, GalL-ISceI-nat::leu2-3,112, ura3-52, lys2-801, mNeonGreen-PAN1, GTS1-mScarlet::hphNT1*	This Study
MSK365	GFP-End3, Ent1-mCherry	*MATα, his3Δ200, GalL-ISceI-nat::leu2-3,112, ura3-52, lys2-801, GFP-END3::natNT2, ENT1-mCherry::hphNT1*	This Study
MSK366	GFP-End3, Ent2-mCherry	*MATα, his3Δ200, GalL-ISceI-nat::leu2-3,112, ura3-52, lys2-801, GFP-END3::natNT2, ENT2-mCherry::hphNT1*	This Study
MSK367	Apl1-GFP, End3-mCherry	*MATα, his3Δ200, leu2-3,112, ura3-52, lys2-801, APL1-EGFP::natNT2, END3-mCherry::hphNT1*	This Study

MSK368	Apl1-GFP, Ent2-mCherry	*MATα, his3Δ200, leu2-3,112, ura3-52, lys2-801, APL1-EGFP::natNT2, ENT2-mCherry::hphNT1*	This Study
MSK369	mNG-Pan1, mSc-Gts1	*MATα, his3Δ200, GalL-ISceI-nat::leu2-3,112, ura3-52, lys2-801, mNeonGreen-PAN1, mScarlet-GTS1*	This Study
MSK372	mNG-Sla2, mSc-Gts1	*MATα, his3Δ200, GalL-ISceI-nat::leu2-3,112, ura3-52, lys2-801, mNeonGreen-SLA2, mScarlet-GTS1*	This Study
MSK386	Gts1-GFP, Sla2-mCherry	*MATα, his3Δ200, leu2-3,112, ura3-52, lys2-801, GTS1-EGFP::HIS3MX6, SLA2-mCherry::hphNT1*	This Study
MSK398	Yap1801-GFP, Gts1-mCherry	*MATα, his3Δ200, leu2-3,112, ura3-52, lys2-801, YAP1801-EGFP::HIS3MX6, Gts1-mCherry::hphNT1*	This Study
MSK420	mTq2-Las17, Sla1-mNG	*MATα, his3Δ200, GalL-ISceI-nat::leu2-3,112, ura3-52, lys2-801, mTurquoise2-LAS17, SLA1-mNeonGreen::kanMX4*	This Study
MSK424	GFP-Gts1	*MATα, his3Δ200, GalL-ISceI-nat::leu2-3,112, ura3-52, lys2-801, sgGFP-GTS1*	This Study
MSK426	mScarlet-Gts1	*MATα, his3Δ200, GalL-ISceI-nat::leu2-3,112, ura3-52, lys2-801, mScarlet-I-GTS1*	This Study
MSK458	Pan1(1-1303)-mTq2, Sla2-mNG, Abp1-mSc	*MATα, his3Δ200, leu2-3,112, ura3-52, lys2-801, PAN1(aa 1-1303)-mTurquoise2::URA3, SLA2-mNeonGreen::kanMX, ABF*	This Study
MSK505	Ent1-LIDLD-mTq2, Sla1-mNG, Abp1-mSc	*MATα, his3Δ200, leu2-3,112, ura3-52, lys2-801, ENT1+D codon-mTurquoise2::hphNT1, SLA1-mNeonGreen::kanMX4, AB*	This Study
MSK511	Ent1-LIDLD-mTq2, Sla2-mNG, Abp1-mSc	*MATα, his3Δ200, leu2-3,112, ura3-52, lys2-801, ENT1+D codon-mTurquoise2::natNT2, SLA2-mNeonGreen::kanMX4, ABI*	This Study
MSK524	Pan1(1-1303)-mTq2, Abp1-mSc	*MATα, his3Δ200, leu2-3,112, ura3-52, lys2-801, PAN1(aa 1-1303)-mTurquoise2::URA3, ABP1-mScarletI::hphNT1*	This Study
MSK526	Ent1-LIDLD-mTq2, Abp1-mSc	*MATα, his3Δ200, leu2-3,112, ura3-52, lys2-801, ENT1+D codon-mTurquoise2::hphNT1, ABP1-mScarlet-I::natNT2*	This Study
MSK528	Las17-GFP, Sla1-mCherry	*MATa, his3Δ200, leu2-3,112, ura3-52, lys2-801, LAS17-EGFP::HIS3MX6, SLA1-mCherry::hphNT1*	This Study
MSK529	Yap1801-GFP, Syp1-mCherry	*MATα, his3Δ200, leu2-3,112, ura3-52, lys2-801, YAP1801-EGFP::HIS3MX6, SYP1-mCherry::hphNT1*	This Study
MSK530	Yap1802-GFP, Syp1-mCherry	*MATα, his3Δ200, leu2-3,112, ura3-52, lys2-801, YAP1802-EGFP::HIS3MX6, SYP1-mCherry::hphNT1*	This Study
MSK531	Apl1-GFP, Syp1-mCherry	*MATα, his3Δ200, leu2-3,112, ura3-52, lys2-801, APL1-EGFP::natNT2, SYP1-mCherry::hphNT1*	This Study
MSK534	mNG-Sla2, mSc-Pan1	*MATα, his3Δ200, GalL-ISceI-nat::leu2-3,112, ura3-52, lys2-801, mNeonGreen-SLA2, mScarlet-I-PAN1*	This Study
MSK535	mNG-Pan1, mSc-Sla2	*MATα, his3Δ200, GalL-ISceI-nat::leu2-3,112, ura3-52, lys2-801, mNeonGreen-PAN1, mScarlet-I-SLA2*	This Study
MSK536	sla2dTHATCH-GFP, Chc1-mCherry	*MATα, his3Δ200, leu2-3,112, ura3-52, lys2-801, sla2(aa 1-765)-EGFP::HISMX6, CHC1-mCherry::hphNT1*	This Study
MSK537	sla2dTHATCH-GFP, Clc1-mCherry	*MATα, his3Δ200, leu2-3,112, ura3-52, lys2-801, sla2(aa 1-765)-EGFP::HISMX6, CLC1-mCherry::hphNT1*	This Study
MSK538	Pan1(1-1050)-GFP, Chc1-mCherry	*MATα, his3Δ200, leu2-3,112, ura3-52, lys2-801, pan1(aa 1-1050)-EGFP::HISMX6, CHC1-mCherry::hphNT1*	This Study
MSK539	Pan1(1-1050)-GFP, Clc1-mCherry	*MATα, his3Δ200, leu2-3,112, ura3-52, lys2-801, pan1(aa 1-1050)-EGFP::HISMX6, CLC1-mCherry::hphNT1*	This Study
MSK540	Pan1(402-1480)-GFP, Chc1-mCherry	*MATα, his3Δ200, leu2-3,112, GalL-ISceI-nat::leu2-3,112, lys2-801, pan1(aa 402-1480)-EGFP::HISMX6, CHC1-mCherry::h*	This Study
MSK541	Pan1(402-1480)-GFP, Clc1-mCherry	*MATα, his3Δ200, leu2-3,112, GalL-ISceI-nat::leu2-3,112, lys2-801, pan1(aa 402-1480)-EGFP::HISMX6, CLC1-mCherry::h*	This Study
MSK542	Yap1801-GFP, Sla2-mCherry	*MATa, his3Δ200, leu2-3,112, ura3-52, lys2-801, YAP1801-EGFP::natNT2, SLA2-mCherry:::hphNT1*	This Study
SL2793	two-hybrid strain PJ964a	*MATa, his3Δ200, leu2-3,112, ura3-52, trp1-901, gal4Δ, ade2, gal80Δ, GAL2-ADE2, LYS2::GAL1-HIS3, met2::GAL7-lacZ*	S. Lemmon Lab
