# Supplementary material for: The protein architecture of the endocytic coat analyzed by FRET microscopy
Source: Mol Syst Biol. 2020 May 13;16(5):e9009. doi: 10.15252/msb.20199009 (PMC7218409; doi:10.15252/msb.20199009)
Supplement: Supplementary file 2 — Expanded View Figures PDF [file MSB-16-e9009-s002.pdf]

Expanded View Figures

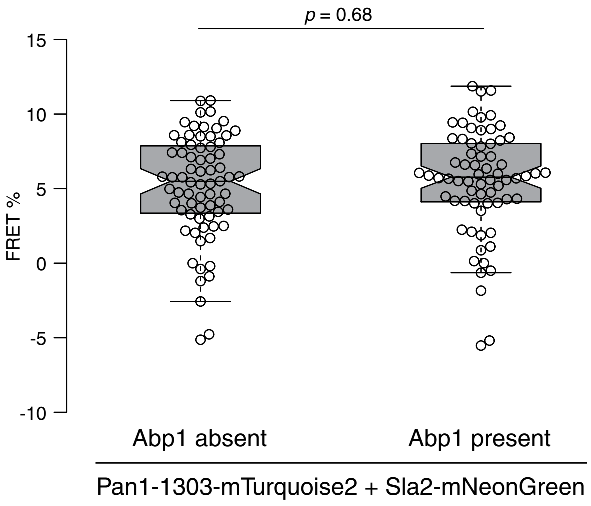

Figure EV1. FRET proximity between Pan1 (1–1303) and Sla2 C-termini does not change during membrane invagination.

FRET values (in %) of 71 or 68 endocytic patches containing or absent of Abp1, respectively, are shown as box plots. Center, top, and bottom lines of box plots show the medians, the 25th, and 75th percentiles of individual datasets, respectively. Whiskers extend 1.5 times the interquartile range from the 25th and 75th percentiles. Notches indicate 95% confidence intervals. Statistical difference was analyzed by Welch's *t*-test.

© 2020 The Authors
