# Supplementary material for: The protein architecture of the endocytic coat analyzed by FRET microscopy
Source: Mol Syst Biol. 2020 May 13;16(5):e9009. doi: 10.15252/msb.20199009 (PMC7218409; doi:10.15252/msb.20199009)
Supplement: Supplementary file 4 — Movie EV1 [file MSB-16-e9009-s004.zip › Movie EV1.docx]

**Movie EV1 - FRET between indicated protein pairs observed as an increase of GFP donor fluorescence after mCherry acceptor photobleaching.**

Fluorescence of GFP FRET donor attached to the indicated protein was observed before and after photobleaching of mCherry FRET acceptor. Five frames of 500-1000 ms exposure acquired before and after photobleaching are shown.
