# Supplementary material for: The protein architecture of the endocytic coat analyzed by FRET microscopy
Source: Mol Syst Biol. 2020 May 13;16(5):e9009. doi: 10.15252/msb.20199009 (PMC7218409; doi:10.15252/msb.20199009)
Supplement: Supplementary file 5 — Movie EV2 [file MSB-16-e9009-s005.zip › Movie EV2.docx]

**Movie EV2 - FRAP of mNeonGreen-tagged Ede1, Apl1, Yap1801, Gts1, Bzz1, Lsb3, Las17, and Vrp1 proteins in LatA-treated wild-type cells.**

Indicated proteins were photobleached at the endocytic sites marked with arrows (at time 0) and their fluorescence recovery was followed for 60 s with 500 ms frame rate.
